# Dental Department of Meharry Medical College

**Published:** 1889-02

**Authors:** 


### Dental Department of Meharry Medical College.

The corner-stone of the building to be used for the Dental Department of Meharry Medical College was laid at Nashville, Tenn., Oct. 23, 1888. The Meharry Medical College, which is now attached to the Central University of Tennessee, is intended to give the colored people of the South a more thorough knowledge of the medical and dental sciences. It is the intention oft he Faculty to add also a Pharmaceutical Department, the need of which has been frequently manifested by calls upon the institution for educated colored pharmacists. With the exception of the Howard School in Washington, which is for white and colored, there is no other medical school for colored people in the United States.

